# Allergic rhinitis and nasal septum deviation in children: cause, consequence, or bidirectional relationship? Insights from the ARHINASD study

**DOI:** 10.3389/fmed.2026.1824028

**Published:** 2026-05-22

**Authors:** Laura Carucci, Rita Nocerino, Elena Cantone, Francesco Berni Canani, Margherita Licito, Luciano La Marca, Margherita Farnetano, Antonio Masino, Franca Oglio, Dario Marziali, Serena Coppola, Roberto Berni Canani

**Affiliations:** 1 Department of Translational Medical Science, University Federico II, Naples, Italy; 2 ImmunoNutritionLab at CEINGE Advanced Biotechnologies, University Federico II, Naples, Italy; 3 Department of Neuroscience, Reproductive Science and Odontostomatology, University Federico II, Naples, Italy; 4 ENT Section, Department of Pharmacy and Health and Nutrition Sciences, University of Calabria, Calabria, Italy; 5 ENT Unit, Annunziata Hospital, Cosenza, Italy; 6 Otorhinolaryngology Unit, Penne and Popoli Hospitals, ASL of Pescara, Pescara, Italy; 7 Internal Medicine Kantonsspital Graubuenden, Chur, Switzerland; 8 European Laboratory for the Investigation of Food-Induced Diseases, University Federico II, Naples, Italy; 9 NutriTechLab, University Federico II, Naples, Italy

**Keywords:** allergic rhinitis, ARIA guidelines, children, nasal cytokines, nasal cytology

## Abstract

**Background:**

Allergic rhinitis (AR) is one of the most common allergic conditions in children. Nasal septum deviation (NSD) is observed in nearly 34% of the pediatric population and is characterized by reduced nasal airflow and mucosal inflammation. While NSD and AR can coexist, the potential relationship between the two conditions remains largely unexplored. Here, we present the baseline data from the Allergic Rhinitis in pediatric subjects with Nasal Septum Deviation (ARHINASD) study, which investigates the relationship between NSD and AR in children.

**Methods:**

A multicenter, prospective study was conducted involving 138 participants aged 6–14 years with (NSD+, *n* = 69) or without NSD (NSD−, *n* = 69). AR diagnosis and sensitization, nasal endoscopy and cytology, nasal fluid cytokines, nasal airflow (nasal peak inspiratory flow, NPIF), and nasal obstruction (visual analog scale, VAS) were evaluated in all participants.

**Results:**

The prevalence of AR was significantly higher in NSD+ children than in NSD− children (66.7% vs. 36.2%; *p* < 0.0001). A multivariable logistic regression analysis showed that NSD was independently associated with AR (aOR 3.51, 95% CI 1.65–7.47; *p* = 0.001), along with a family history of allergy (aOR 2.49, 95% CI 1.01–6.16; *p* = 0.048). NSD+ children experienced more severe AR, with higher VAS scores, median (interquartile range, IQR) [7(3.5) vs. 2(4), *p* < 0.05] and lower NPIF values [40(30) vs. 60(40) L/min, *p* < 0.05]. NSD+ children had higher eosinophil counts and increased levels of IL-4, IL-5, and eosinophil cationic protein (ECP) in the nasal fluid compared to NSD− children (*p* < 0.05).

**Conclusion:**

The findings suggest a higher prevalence of AR among NSD children. NSD may negatively influence the disease course of AR. Early identification and management of NSD may improve AR outcomes and reduce the overall disease burden. Longitudinal studies are needed to confirm causality and to explore preventive strategies.

## Introduction

Allergic rhinitis (AR) is one of the most prevalent chronic allergic conditions in the pediatric population, affecting up to 25% of children worldwide and having a significant socioeconomic impact (1–3). It is characterized by an IgE-mediated hypersensitivity reaction to inhaled environmental allergens, resulting in nasal congestion, rhinorrhea, sneezing, nasal itching, and postnasal drip. The pathogenesis of AR is multifactorial and involves a complex interplay between genetic predisposition and environmental exposures, including a family history of allergy, early-life exposure to indoor allergens (e.g., dust mites and pet dander), tobacco smoke exposure, and urban living environments (4, 5). Although these factors are well established, emerging evidence suggests that anatomical factors may also influence the development or exacerbation of allergic airway diseases (6).

Nasal septum deviation (NSD) can be observed in up to 34% of children and adolescents and typically results from congenital factors, birth trauma, or postnatal injuries (6, 7). NSD can lead to uneven airflow in the nasal passages, which often results in the obstruction of one or both passages. This altered airflow pattern may contribute to mucosal drying, stasis of secretions, and localized inflammation, along with increased cytokine levels (8). As a result, these changes may impair the mucociliary clearance and potentially facilitate the risk of allergen deposition and sensitization of the nasal mucosa (9).

Although NSD and AR can coexist, the directionality of this association remains uncertain in the pediatric population (6, 10, 11).

The Allergic Rhinitis in pediatric subjects with Nasal Septum Deviation (ARHINASD) project was launched to investigate the potential role of NSD in influencing the occurrence and disease course of AR among children. In this article, we present the baseline data from a pediatric population evaluated at different tertiary centers in Italy.

## Methods

### Study design

A multicenter, prospective observational study was conducted involving three Italian tertiary centers: the University of Naples “Federico II” (Department of Translational Medical Science and Department of Neuroscience, Reproductive Science and Odontostomatology) (Coordinating Center); the University of Calabria (Department of Pharmacy, Health and Nutrition Sciences); and the Otorhinolaryngology Unit, Penne Hospital, ASL of Pescara. From December 2023 to July 2024, all consecutive children aged ≥6 and ≤14 years, with or without NSD, who attended the participating centers for screening programs or minor surgery procedures not involving the respiratory tract, were evaluated for the study. The exclusion criteria were as follows: age ≤6 or ≥14 years; non-Caucasian ethnicity; concomitant presence at baseline of infectious diseases; chronic systemic diseases; malignancies; immunodeficiencies; obesity; autoimmune diseases; celiac disease; metabolic and genetic diseases; cystic fibrosis or other forms of primary pancreatic insufficiency; malformations or previous major surgery procedures involving the gastrointestinal, cardiovascular, urinary, or respiratory tracts; psychiatric or neurological diseases; eosinophilic gastrointestinal disorders; use of antibiotics, topical or systemic antihistamines, or corticosteroids within 7 days before enrollment; nasal trauma within the 3 years preceding enrollment; and a history of prior nasal surgery.

### Study outcomes

The primary study outcome was to evaluate the rate of AR diagnosis in participants with or without NSD. Additionally, the study aimed to evaluate the impact of NSD on the disease course of AR, nasal inflammation, and cytology in patients with NSD (Group NSD+) and those without NSD (Group NSD−).

### Sample size calculation

Based on our previous observational data, we expected an AR prevalence of 65% in children with NSD. To detect a difference of 0.30 in the proportion of AR between the NSD+ and NSD− groups, we calculated that 69 participants per group (Group NSD+ and Group NSD−) were required, assuming an alpha level of 0.05 and a power of 0.95 (Pearson’s chi-squared test) (Stata 14.0, Stata Corp, College Station, TX, USA).

### Statistical analysis

The Kolmogorov–Smirnov test was used to assess the distribution of continuous variables. Normally distributed variables were reported as means and standard deviations (SDs), whereas non-normally distributed variables were reported as medians and interquartile ranges (IQRs), with minimum and maximum values when appropriate. Categorical variables were reported as the number and proportion of participants with the characteristic of interest. Between-group comparisons of continuous variables were performed using the independent samples *t-*test or the Mann–Whitney *U* test, as appropriate. Comparisons of categorical variables were performed using the *χ*^2^ test or Fisher’s exact test, as appropriate. The correlation between nasal peak inspiratory flow (NPIF) and the visual analog scale (VAS) score was assessed using Spearman’s correlation coefficient. Cytokine levels were also compared after stratification according to AR status using the Mann–Whitney *U* test. Within the NSD+ group, exploratory comparisons across Mladina subtypes were performed using the Kruskal–Wallis test for continuous variables. For categorical nasal cytology variables based on the Mladina subtype, the *χ*^2^ test was used, and Monte Carlo exact tests or Fisher–Freeman–Halton tests were applied when expected cell counts were small. A multivariable logistic regression model was used, incorporating all clinically relevant covariates (sex, age at enrolment, a family history of allergy, presence of pets, passive smoke exposure, mold exposure, and study group).

All tests were two-sided, and a *p*-value of <0.05 was considered statistically significant. Statistical analyses were performed using SPSS for Windows (SPSS Inc., version 23.0, Chicago, IL, USA).

### Study procedures

All consecutive participants considered eligible for the study were evaluated for the presence of exclusion criteria. Written informed consent was obtained, and nasal endoscopy was performed to accurately assign participants to the NSD+ and NSD− groups by a research team (RT) composed of pediatricians, otolaryngologists, and allergists. NSD was classified according to Mladina’s (12) score. Once assigned to one of the two groups, each enrolled participant underwent an anamnestic, demographic, and full clinical evaluation by a multidisciplinary team (MT) composed of pediatricians, allergists, otolaryngologists, and nurses, who were unaware of the outcomes of the study. Demographic, anamnestic (such as sex, age, a family history of allergy, exposure to passive smoke, molds, and pets, as well as the presence and recurrence of rhinitis symptoms including nasal congestion, rhinorrhea, sneezing, and nasal itching and age at symptom onset), and clinic data were collected anonymously for each enrolled participant.

**Figure 1:**
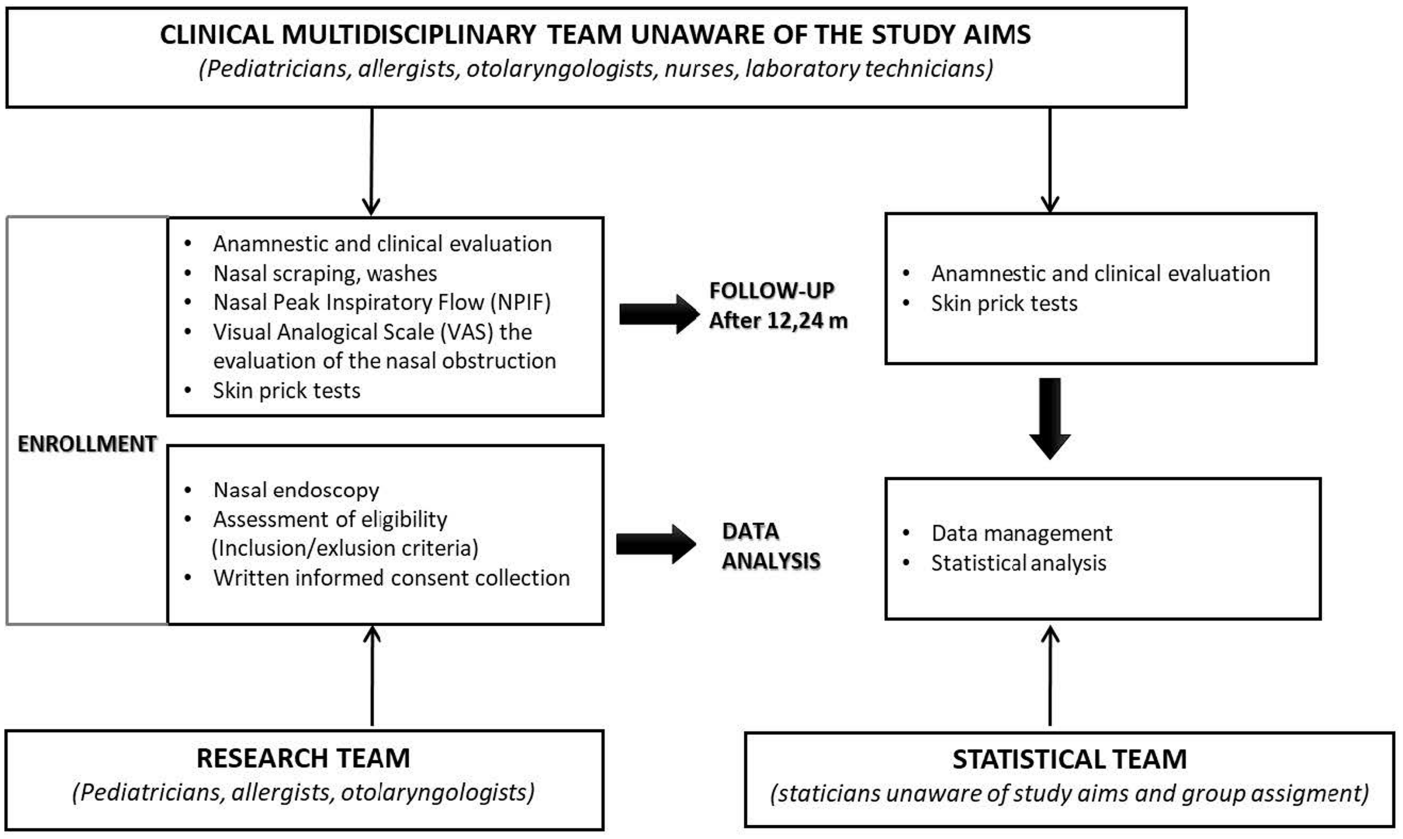
The ARHINASD study design.

After the clinical evaluation, each participant underwent nasal scraping for the assessment of nasal cytology, nasal wash for the measurement of inflammatory cytokines, NPIF testing for the assessment of nasal flow, and a VAS assessment for the evaluation of nasal obstruction. Finally, skin prick tests (SPTs) for inhalant allergens were performed to evaluate allergic sensitization, as previously described (13). The diagnosis and severity of AR were assessed by pediatricians and allergists according to the current allergic rhinitis and its impact on asthma (ARIA) guidelines (14). The total nasal symptom score (TNSS) was also recorded. Laboratory analyses were performed in an anonymous manner by laboratory technicians who were unaware of the study’s aims and group assignment. All data were collected anonymously and entered into the study database using a single data entry method by the same researcher. A clinical trial monitor reviewed the clinical forms for completeness, clarity, consistency, and accuracy. The study database was cleaned according to standard procedures and was locked before statistical analysis by the statistical team. Then, the statistical team, unaware of the study’s aims, reviewed the study database and performed data cleaning and verification according to standard procedures. After the baseline assessment, participants without AR were invited to annual visits for 24 months to monitor the possible occurrence of AR. At each visit, clinical assessment and SPT were performed, as previously described. The study design is presented in Figure 1.

#### Nasal endoscopy

Nasal endoscopy was performed to assess the presence and type of NSD using a pediatric flexible endoscope equipped with an optical fiber system and a connected mini-camera (Olympus®). The findings were classified according to Mladina’s (12) classification system. During the procedure, the participant was seated on a chair, and the endoscope was introduced into the nasal cavities without any anesthetic preparation.

#### Nasal washes and cytokine evaluation

Nasal wash was performed using 10 mL of saline solution. The fluid was collected in 15 mL polypropylene tubes. The nasal lavage was immediately centrifuged at 2000 x *g* for a few min to remove larger cells and cellular debris. Subsequent spins at 10,000 x *g* for 30 min were performed, followed by a final high-speed spin at 100,000 × *g* for 90 min. At the end of centrifugation, the supernatant was collected and stored in new tubes at −20 °C until use, and the resuspended pellet was typically stored at −80 °C until analysis. The assessment of IL-4, IL-5, IL-13, tumor necrosis factor-alpha (TNF-α), and IFN-γ levels was performed using the Bio-Plex Pro Human Cytokine GI 27-Plex Panel (Bio-Rad, Hercules, CA, USA) on the Luminex Multiplex System (LX 200; Luminex Co., Austin, TX, USA) according to the manufacturer’s instructions. Cytokine levels were expressed in pg/mL.

#### Nasal scraping and cytology

Nasal cytology for evaluation of nasal mucosal cells was assessed by performing nasal scraping. Scraping was performed on the middle portion of the inferior turbinate using a cotton bud. The collected cellular material was spread onto a small glass slide and air-dried. It was then stained with May-Grunwald Giemsa stains. Finally, the analysis was conducted using an optical microscope (Zeiss © Primo Star), with an objective capable of magnification up to 100×.

#### NPIF test

The NPIF test was performed to evaluate nasal peak flow in liters per min during maximal nasal inspiration. Briefly, NPIF was measured using a nasal peak inspiratory flow meter (Clement Clark International, Mountain Ash, UK) and expressed in liters per min (L/min). The patient was seated comfortably and wore a face mask that was held firmly against the face by one of the researchers, applying sufficient pressure to prevent air leakage. The child was asked to blow the nose before performing the test, to take 10 calm breaths, and then to perform a maximal nasal expiration. The child was instructed to perform a maximal inspiratory effort through the nose with the lips closed. The manoeuvre was repeated three times, and the highest of the three measurements was recorded, with a variation of up to 10%.

#### VAS

The VAS, using a faces rating scale, was used to assess nasal obstruction. The scale ranged from 0, represented by a happy face (no obstruction), to 10, represented by a crying face (most severe obstruction).

#### Total nasal symptom score

Nasal symptoms were also evaluated using the TNSS, a validated composite measure widely used in AR clinical trials (15, 16). The TNSS is a widely used composite score assessing nasal congestion, rhinorrhea, nasal itching, and sneezing (0–3 for each symptom; total range 0–12), with higher scores indicating greater symptom severity. Scores ≤5 are generally considered consistent with mild rhinitis in clinical research settings, while scores >6 indicate moderate/severe symptoms.

#### Skin prick tests

SPTs for aeroallergens were performed using aeroallergen extracts, (including birch, cypress, grasses, mugwort, olive, *Parietaria officinalis*, ragweed, dust mites, cat and dog epithelium, *Alternaria*, *Cladosporium*, and *Aspergillus*) (Lofarma S.p.A., Milan, Italy), as previously described (13).

## Results

### Study participants

**Figure 2:**
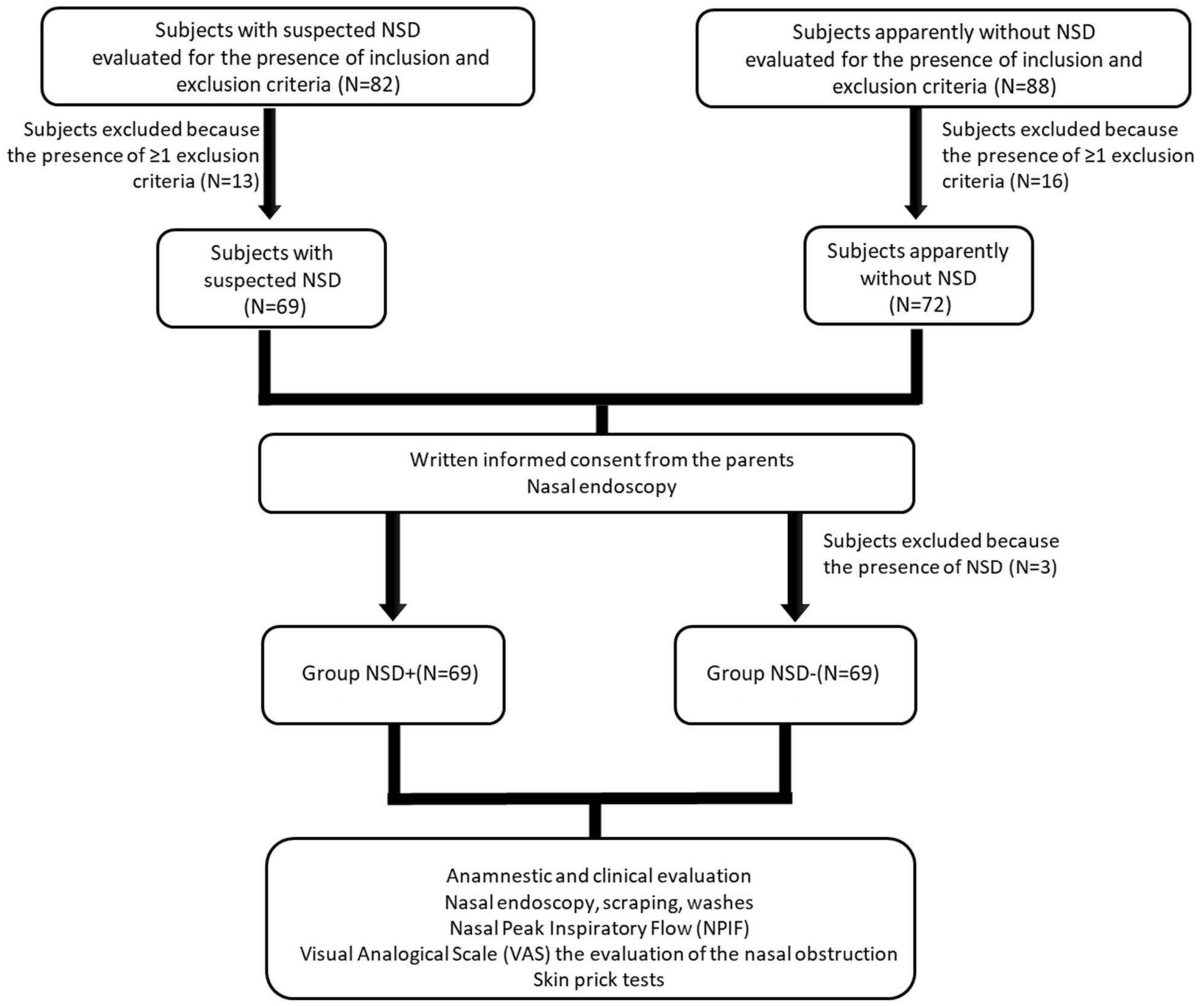
Flow of participants. NSD, nasal septum deviation.

From January 2023 to July 2024, a total of 170 consecutive participants (82 children with suspected NSD and 88 children apparently without NSD) were evaluated for the study. Among those with suspected NSD, 13 were excluded due to the presence of at least one exclusion criterion (3 had recent nasal trauma, 7 had recently used antibiotics, and 3 had recently used topical or systemic corticosteroids/antihistamines). Among participants apparently without NSD, 16 were excluded because of the presence of at least one exclusion criterion (1 had neuropsychiatric condition, 1 had eosinophilic gastrointestinal disorders, 6 had recently used antibiotics, 8 had recently used recent topical or systemic corticosteroids/antihistamines). Therefore, a total of 141 participants were included in the study: 69 in the group with suspected NSD and 72 in the group without NSD. After nasal endoscopy, NSD was confirmed in all 69 participants with suspected NSD, who were therefore enrolled in the NSD+ group. Conversely, NSD was not confirmed in 69 of 72 participants apparently without NSD. Therefore, 3 participants were subsequently excluded, and 69 were enrolled in the NSD− group (Figure 2).

The main anamnestic and clinical features of the two study populations were similar and are reported in Table 1. According to the Mladina score, no patient presented with type 7 deviation (Supplementary Table 1).

**Table 1:** Main anamnestic and clinical features of the study population.

Features	Group NSD+	Group NSD−	*p*
*N*	69	69	ns
Male, *n* (%)	42 (60.9)	37 (53.6)	ns
Age at enrolment, years (IQR)	10 (5; 6–14)	8 (4; 6–14)	ns
Weight, kg (IQR)	36.6 (20.1)	33.6 (15.2)	ns
Height, cm (IQR)	143.5 (24.9)	132.8 (26.2)	ns
Body mass index (IQR)	17.6 (4.5)	17.5 (5.5)	ns
Family history of allergy, *n* (%)	51 (73.9)	56 (81.2)	ns
Passive smoke exposure, *n* (%)	19 (27.5)	28 (40.6)	ns
Mold exposure, *n* (%)	8 (11.6)	15 (21.7)	ns
Presence of pets, *n* (%)	16 (23.2)	20 (29)	ns

NSD, nasal septum deviation; IQR, interquartile range.

### Main study outcome

The prevalence of AR was significantly higher in the NSD+ group (0.67, 95%CI 0.55–0.78) compared to the NSD– group (0.36, 95%CI 0.25–0.48) (*p <* 0.001). The multivariable logistic regression analysis showed that NSD was independently associated with AR (aOR 3.51, 95% CI 1.65–7.47; *p* = 0.001) after adjusting for age, sex, a family history of allergy, and environmental exposures. A family history of allergy was also independently associated with AR (aOR 2.49, 95% CI 1.01–6.16; *p* = 0.048), while no significant associations were observed for the other variables included in the model. Supplementary Table 2 reports the Mladina score in participants with and without AR.

In participants with AR, the distribution of causative allergens was similar in both groups (Supplementary Table 3). However, when comparing AR patients in the NSD+ group to those in the NSD− group, the NSD+ group showed a higher rate of sensitization to multiple allergens (0.85, 95%CI 0.74–0.96 vs. 0.64, 95%CI 0.44–0.84; *p* < 0.05).

### Secondary study outcomes

**Figure 3:**
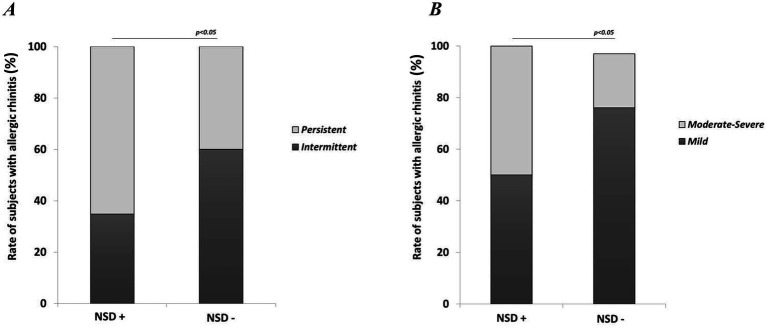
Frequency **(A)** and severity **(B)** of allergic rhinitis between the study groups. NSD+: group of participants with nasal septum deviation. NSD−: Group of participants without nasal septum deviation.

To assess the impact of NSD on the disease course of AR, the frequency (Figure 3A) and severity (Figure 3B) of AR symptoms were evaluated following the ARIA guidelines (14). AR patients in the NSD+ group showed a higher prevalence of persistent symptoms (0.65, 95%CI 0.51–0.79 vs. 0.40, 95%CI 0.19–0.61, *p* < 0.05) and more severe symptoms (with a moderate-to-high level) (0.50, 95%CI 0.35–0.65 vs. 0.24, 95%CI 0.06–0.42, *p* < 0.05) compared to those in the NSD− group (Figure 3). Similarly, AR participants in the NSD+ group showed higher TNSS values compared to those in the NSD− group (mean ± SD: 6.65 ± 2.20 vs. 4.56 ± 1.58; *p* < 0.001), suggesting that the presence of NSD could also influence the severity of non-mechanical AR symptoms.

**Figure 4:**
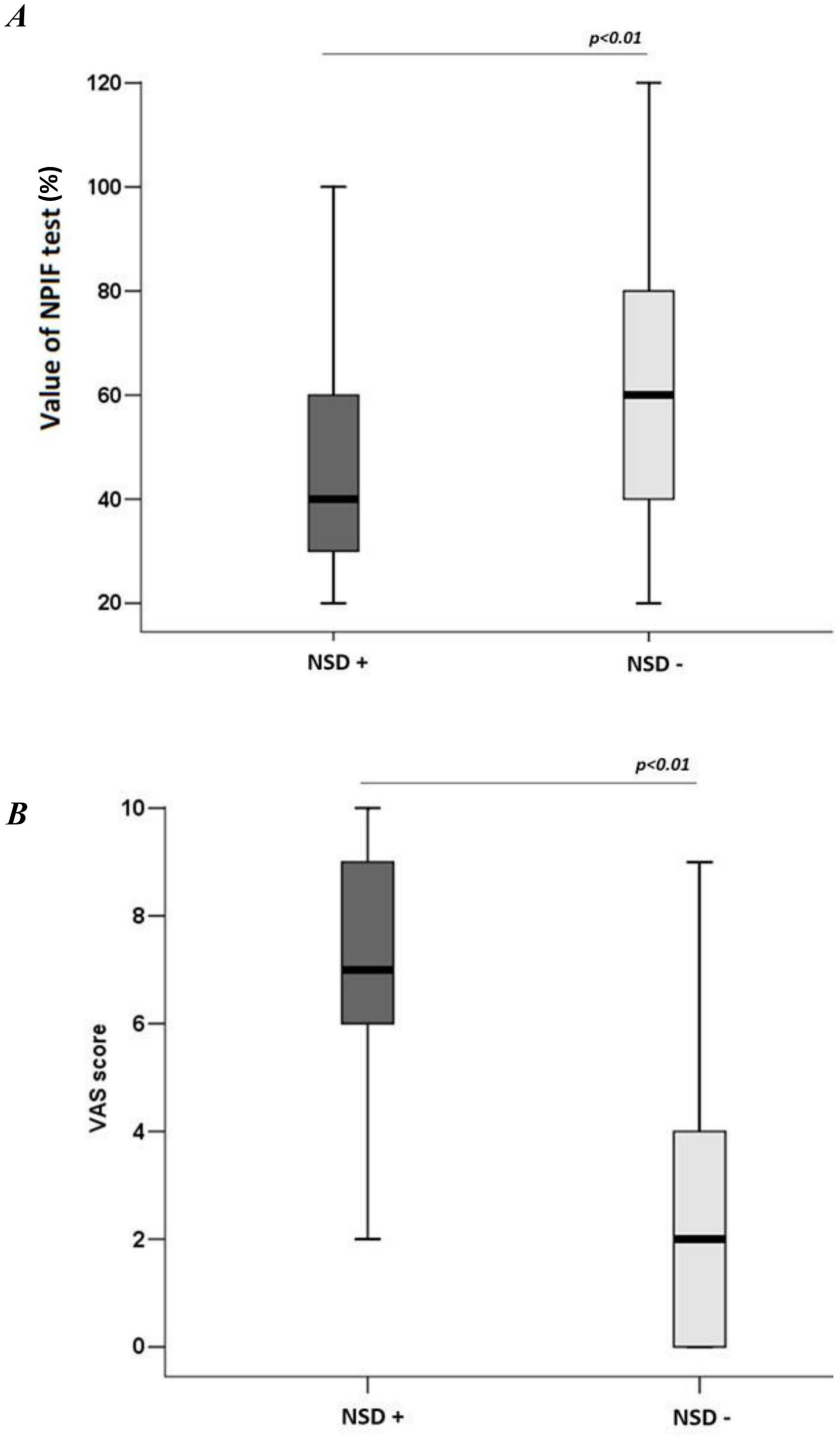
Median values of the nasal peak inspiratory flow test **(A)** and nasal obstruction symptoms assessed using the VAS **(B)** in the two study groups. NPIF, nasal peak inspiratory flow; VAS, visual analog scale.

The role of NSD in influencing the disease course of AR was also evaluated by assessing nasal obstruction using both objective and subjective methods. The NPIF test showed a significantly lower median value (IQR) in the NSD+ group [40(30) vs. 60(40) in the NSD− group; *p* < 0.05] (Figure 4A). Similarly, the VAS score was significantly higher in the NSD+ group [7(3.5) vs. 2(4) in the NSD− group; *p* < 0.05] (Figure 4B), confirming greater severity of AR, as assessed according to the ARIA guidelines (14). Furthermore, a significant negative correlation between NPIF values and the VAS score was observed (*r* = −0.427, *p <* 0.01) in all study participants. When stratified by NSD status, this correlation was also confirmed in the NSD+ group (*r* = −0.363, *p <* 0.01).

**Figure 5:**
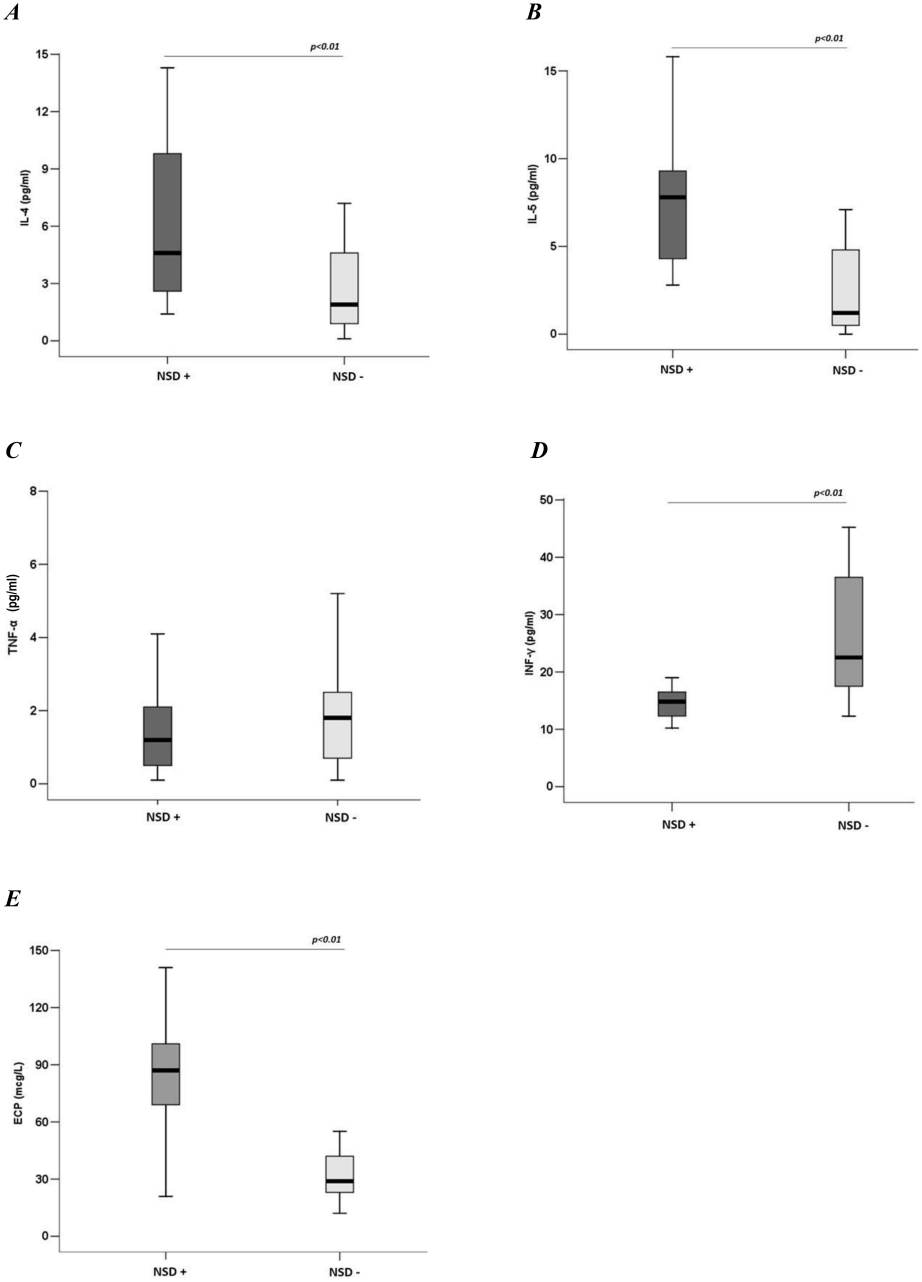
Cytokine levels in nasal washes collected from participants in the two study groups. **(A)** Levels of interleukin-4 (IL-4); **(B)** levels of interleukin-5 (IL.5); **(C)** levels of transforming growth factor-alpha (TNF-α); **(D)** levels of interferon-gamma (IFN-γ); **(E)** levels of eosinophil cationic protein (ECP).

The possible influence of NSD on nasal inflammation was assessed through the evaluation of cytokine levels in nasal washes and nasal cytology. Significantly higher levels of IL-4, IL-5, and eosinophil cationic protein (ECP) and significantly lower levels of IFN-γ were observed in the NSD+ group compared to the NSD− group (Figure 5). In the stratified analysis, AR−/NSD+ participants showed higher IL-4 and IL-5 levels and lower IFN-γ levels compared to AR−/NSD− participants. Among participants with AR, those in the NSD+ group showed higher IL-4 and IL-5 levels compared to the NSD− group, while no significant difference in IFN-γ was observed. These stratified data are reported in Supplementary Table 4. Nasal cytology revealed a significantly higher presence of ciliated cells, goblet cells, neutrophils, eosinophils, and mast cells in the NSD+ group. A similar number of bacteria was found between the study groups. Furthermore, within the NSD+ group, a significantly higher number of ciliated cells, goblet cells, eosinophils, and mast cells was detected in AR patients compared to those without AR (Table 2). Among participants without AR, those in the NSD+ group showed higher levels of all evaluated cell types, suggesting a potential role of NSD in inducing a pro-inflammatory state. Spores and lymphocytes were not detected in any study participants.

**Table 2:** Evaluation of nasal cytology in the study groups.

Type of cells	Group NSD+ (*n* = 69)		*p*	Group NSD− (*n* = 69)		*p*
	AR+ (*n* = 46)	AR− (*n* = 23)		AR+ (*n* = 25)	AR− (*n* = 44)	
Ciliated cells, *n* (%)						
+	32 (69.6)	20 (86.9)	ns	20 (80)	30 (68.2)	ns
++	10 (21.7)	0 (0)	<0.05	0 (0)	0 (0)	ns
Goblet cells, *n* (%)						
+	33 (71.7)	9 (39.1)	<0.05	15 (60)	5 (11.4)	<0.05
Neutrophils, *n* (%)						
+	14 (30.4)	3 (13)	ns	3 (12)	4 (9.1)	ns
+++	0 (0)	0 (0)	ns	1 (4)	0 (0)	ns
Eosinophils, *n* (%)						
+	42 (91.3)	6 (26.1)	<0.05	10 (40)	0 (0)	<0.05
Mast cells, *n* (%)						
+	33 (71.4)	7 (30.4)	<0.05	13 (52)	0 (0)	<0.05
Bacteria, *n* (%)						
+	0 (0)	0 (0)	ns	0 (0)	4 (9.1)	ns
++	0 (0)	2 (8.7)	ns	0 (0)	3 (6.8)	ns

AR, allergic rhinitis; NSD, nasal septum deviation.

Finally, exploratory analyses within the NSD+ group revealed no differences across Mladina subtypes for IL-4, IL-5, IL-13, TNF-α, ECP, and IFN-γ. Similarly, no significant differences were observed across Mladina subtypes for ciliated cells, goblet cells, neutrophils, eosinophils, mast cells, and bacteria. These subtype-specific analyses according to the Mladina classification should be interpreted cautiously because they were exploratory and some Mladina subtypes included a limited number of participants (Supplementary Table 2).

## Discussion

Allergic rhinitis is one of the most common allergic conditions in children (1–3), and it strongly contributes to the economic burden on healthcare systems and families, with an estimated annual total cost of €9.9 billion in European countries (17). These costs are further increased by the fact that AR is a major risk factor for the development of asthma (18) and that it negatively impacts the control of concomitant childhood asthma, increasing the risk of hospitalization, medical visits, and use of asthma controller drugs (19, 20). Although it has been suggested that AR pathogenesis depends on a complex interplay between genetic and environmental factors and that anatomical abnormalities could worsen AR symptoms (6), the role of NSD as a potential driver of the occurrence and disease course of AR in the pediatric population is still poorly defined.

The preliminary results of the ARHINASD study presented in this article suggest a plausible association between NSD and AR in the pediatric population. Our findings demonstrated that children with NSD showed a higher rate of AR (67% vs. 36%, *p* < 0.001) and that NSD increased the risk of developing AR by 3.5 times. In addition, our study confirms that having a first-degree family member affected by allergy (i.e., a family history of allergy) is an additional risk factor for AR occurrence, as previously demonstrated (21). The hypothesis is that anatomical alterations, by influencing airflow dynamics, impair mucociliary clearance and prolong allergen contact with the nasal epithelium, thereby facilitating allergic sensitization and AR occurrence and perpetuating local inflammation, with a negative impact on the clinical course of AR when already present (22). To support this hypothesis, we comparatively evaluated Th2 and Th1 cytokine levels in the nasal fluid of participants in the NSD + and NSD- groups. Our findings demonstrated that participants with NSD had higher levels of Th2 inflammation, specifically IL-4 and IL-5, along with increased ECP, which is a marker of eosinophilic activation (23). Conversely, IFN-γ levels were significantly lower in the NSD+ group, suggesting the downregulation of Th1-mediated cytokines, especially in participants affected by AR. In addition, the role of NSD in facilitating inflammation was also supported by the finding that AR-/NSD+ children showed significantly higher levels of IL-4 and IL-5 compared to AR-/NSD− children, while IFN-γ levels were significantly lower. In contrast, among participants with AR, IL-4 and IL-5 levels remained higher in NSD+ participants, while IFN-γ levels did not significantly differ between the groups.

Accordingly, a recent study comparing nasal cytokine levels before and after septoplasty showed a significant reduction in IL-4 levels after nasal corrective surgery, supporting our findings regarding the potential role of NSD in inducing Th2-skewed inflammation (8). Longitudinal studies evaluating the incidence of AR in these categories of participants are needed. The role of NSD in inducing local Th2 inflammation was also confirmed by the nasal cytology results, showing that children with NSD, especially those with concomitant AR, had significantly higher levels of goblet cells, neutrophils, eosinophils, and mast cells compared to children without NSD. Data on nasal cytology in children with NSD, with or without AR, are lacking, which makes it difficult to compare our findings with previous studies.

Another clinical finding that could suggest the potential role of NSD as a driver of AR occurrence and severity is the spectrum of allergen sensitization (e.g., dust mites, pollens, and molds), which showed a higher prevalence of polysensitization in participants with NSD (70.9% vs. 29.1%, *p* < 0.05). This may suggest that persistent inflammation with longer period of nasal mucosal impairment with prolonged exposure to inhalant allergens could increase the risk of multiple sensitization to aeroallergens. Prospective assessment is necessary to confirm this hypothesis. Furthermore, polysensitization has been associated with more severe and persistent AR, which may partly explain the clinical differences observed in our study (24). In our cohort, AR patients in the NSD+ group showed higher rates of persistent AR (65.2% vs. 40%) and greater symptom severity (moderate-to-severe AR in 50% vs. 24%), as assessed according to the ARIA guidelines and the TNSS, as previously reported (6, 15, 16).

Functionally, this was reflected in significantly reduced NPIF values [40(30) vs. 60(40) L/min] and higher scores on the VAS for nasal obstruction [7(3.5) vs. 2(4)], both of which showed a strong inverse correlation (*r* = −0.427, *p* < 0.01), as previously reported (25, 26). These objective and subjective findings further support the clinical relevance of impaired nasal airflow in children with NSD and underscore the role of anatomical impairment in symptom perception and disease burden (6). In addition, the higher TNSS observed in NSD+ children with AR suggests that the increased symptom burden is not solely explained by structural nasal narrowing but also reflects enhanced allergic inflammation.

These findings may have relevant implications for clinical practice and could pave the way for innovative strategies for the prevention and treatment of these conditions. Indeed, children presenting with NSD, particularly those with a positive family history of allergies, the presence of other allergic comorbidities, and/or persistent respiratory symptoms, may benefit from an allergy evaluation to promptly treat nasal inflammation, possibly reduce the risk of AR occurrence, prevent subsequent polysensitization, and lessen the frequency and severity of symptoms. Consequently, this may reduce the need for drugs and lower the overall costs associated with the management of AR. Concomitantly, children with AR who present with persistent nasal symptoms or an inadequate response to standard allergy therapies should be evaluated for underlying anatomical abnormalities, such as NSD, which may worsen symptoms, increase the use of drugs and the risk of polysensitization, with a consequent negative impact on healthcare costs and QoL.

Our study is strengthened by its multidisciplinary and multicenter design, strict inclusion and exclusion criteria, and comprehensive methodological approach. All assessments, including nasal cytology, cytokine analysis, functional nasal airflow measurements, and allergy testing, were conducted using standardized protocols and blinded evaluation, enhancing the internal validity and reproducibility of the results. In addition, one of the key strengths of this study is the integrated evaluation of inflammatory and cellular responses alongside clinical features, which adds greater value to the data obtained.

The limitations of the study include the age range and the ethnic homogeneity, which limit the generalizability of our findings to broader pediatric populations, as well as the lack of a participating center from Northern Italy. Another limitation is that the present analysis represents the baseline evaluation of the ongoing prospective ARHINASD study; therefore, although it reveals a strong association between NSD and AR, it cannot definitively establish whether NSD precedes the development of allergic sensitization. Once available, data on the prospective assessment of the ARHINASD study will clarify the temporal and possibly bidirectional relationship between structural changes and allergic inflammation.

## Conclusion

In conclusion, the ARHINASD study suggests that NSD, by altering nasal airflow, increasing local inflammation and cellularity, and promoting a pro-allergic inflammatory milieu, could facilitate the occurrence of AR and negatively influence the progression of the disease. These results advocate for an integrated clinical approach that considers both anatomical and immunological factors in the evaluation and management of children with NSD; indeed, early identification of NSD, coupled with targeted prevention and management strategies, may reduce AR incidence and improve treatment outcomes. Further longitudinal studies are needed to confirm these associations and explore potential innovative preventive strategies for AR.

## Data Availability

The raw data supporting the conclusions of this article will be made available by the authors, without undue reservation.
