# Correction: Allergic rhinitis and nasal septum deviation in children: cause, consequence, or bidirectional relationship? Insights from the ARHINASD study

**DOI:** 10.3389/fmed.2026.1891726

**Published:** 2026-06-04

**Authors:** Frontiers Production Office

**Affiliations:** Frontiers Media SA, Lausanne, Switzerland

**Keywords:** allergic rhinitis, ARIA guidelines, children, nasal cytokines, nasal cytology

In the published article, the title of this article was erroneously given as:

“*Integrating AI into undergraduate medical education: an exploration of learner-centered approaches through AI”*

The correct title of the article is:

“*Allergic rhinitis and nasal septum deviation in children: cause, consequence, or bidirectional relationship? Insights from the ARHINASD study”*

The original version of this article has been updated.

