# Supplementary material for: Allergic rhinitis and nasal septum deviation in children: cause, consequence, or bidirectional relationship? Insights from the ARHINASD study
Source: Front Med (Lausanne). 2026 May 22;13:1824028. doi: 10.3389/fmed.2026.1824028 (PMC13277362; doi:10.3389/fmed.2026.1824028)
Supplement: Supplementary file 1 [file Data_Sheet_1.docx]

**Supplementary Table 1**. **Classification of nasal septum deviation according to MLADINA score in Group NSD+.**

|  | **Group NSD +** |
| --- | --- |
| **N.** | 69 |
| **MLADINA score** |  |
| ***-type 1, n (%)*** | 20 (29) |
| ***- type 2, n (%)*** | 8 (11.6) |
| ***- type 3, n (%)*** | 6 (8.7) |
| ***- type 4, n (%)*** | 13 (18.8) |
| ***- type 5, n (%)*** | 10 (14.5) |
| ***- type 6, n (%)*** | 12 (17.4) |
| ***- type 7, n (%)*** | 0 |

*NSD: nasal septum deviation*

**Supplementary Table 2**. **Classification of nasal septum deviation according to MLADINA score in patients in Group NSD + stratified for allergic rhinitis presence.**

|  | **AR -** | **AR +** | ***p*** |
| --- | --- | --- | --- |
| **N.** | 23 | 46 |  |
| **Score MLADINA** |  |  |  |
| ***-type 1, n (%)*** | 10 (43.5) | 10 (21.7) | *ns* |
| ***- type 2, n (%)*** | 4 (17.4) | 4 (8.7) | *ns* |
| ***- type 3, n (%)*** | 2 (8.7) | 4 (8.7) | *ns* |
| ***- type 4, n (%)*** | 3 (13) | 10 (21.7) | *ns* |
| ***- type 5, n (%)*** | 2 (8.7) | 8 (17.4) | *ns* |
| ***- type 6, n (%)*** | 2 (8.7) | 10 (21.7) | *ns* |
| ***- type 7, n (%)*** | 0 | 0 | *-* |

*NSD: nasal septum deviation; AR: allergic rhinitis*

**Supplementary Table 3**. **Skin Prick Test positivity for allergens responsible of allergic rhinitis.**

|  | **Group NSD +** | **Group NSD -** | ***p*** |
| --- | --- | --- | --- |
| **N.** | 46 | 25 |  |
| **Mold, n (%)** | 11 (23.9) | 6 (24) | *ns* |
| **Olive, n (%)** | 8 (17.4) | 7 (28) | *ns* |
| **Grasses, n (%)** | 20 (43.5) | 8 (32) | *ns* |
| **Mugwort/Ragweed, n (%)** | 6 (13) | 2 (8) | *ns* |
| **Dog/cat epithelium, n (%)** | 9 (19.6) | 2 (8) | *ns* |
| **Dust mites, n (%)** | 34 (73.9) | 18 (72) | *ns* |
| **Parietaria, n (%)** | 17 (37) | 7 (28) | *ns* |
| **Birch, n (%)** | 2 (4.3) | 1 (4) | *ns* |

*NSD: nasal septum deviation*

**Supplementary Table 4. Stratified analysis of nasal cytokine levels according to NSD and AR status**

| **Cytokine** | **NSD−AR− (n=44)** | **NSD+AR− (n=23)** | **p value** | **NSD−AR+ (n=25)** | **NSD+AR+ (n=46)** | **p value** |
| --- | --- | --- | --- | --- | --- | --- |
| IL-4, median (IQR), pg/mL | 1.30 (1.27) | 2.10 (1.00) | <0.001 | 5.50 (2.45) | 8.70 (5.88) | 0.002 |
| IL-5, median (IQR), pg/mL | 0.60 (0.88) | 3.50 (1.10) | <0.001 | 5.20 (0.80) | 8.55 (2.40) | <0.001 |
| IFN-γ, median (IQR), pg/mL | 34.65 (15.73) | 11.20 (3.90) | <0.001 | 15.40 (3.00) | 15.20 (3.00) | 0.522 |

*Data are reported as median (IQR). P values were obtained using the Mann-Whitney U test for pairwise comparisons between NSD+ and NSD− subjects within each AR status*
